# Supplementary material for: Analysis of SARS-CoV-2 antibody seroprevalence in Northern Ireland during 2020–2021
Source: Heliyon. 2024 Jan 10;10(2):e24184. doi: 10.1016/j.heliyon.2024.e24184 (PMC10830527; doi:10.1016/j.heliyon.2024.e24184)
Supplement: Multimedia component 1 [file mmc1.docx]

**Analysis of SARS-CoV-2 antibody seroprevalence in Northern Ireland during 2020-2021 - Supplementary material**

Michelle K Greene^1^, Peter Smyth^1^, Andrew English^2,3^, Joseph McLaughlin^2^, Magda Bucholc^4^, Janice Bailie^5^, Julie McCarroll^5^, Margaret McDonnell^6^, Alison Watt^7^, George Barnes^8^, Mark Lynch^9^, Kevan Duffin^10^, Gerard Duffy^11^, Claire Lewis^12^, Jacqueline A James^1,12,13^, Alan W Stitt^14^, Tom Ford^15^, Maurice O’Kane^9^, Taranjit Singh Rai^2^, Anthony J Bjourson^2^, Christopher Cardwell^16^, J Stuart Elborn^14^, David S Gibson^2^, Christopher J Scott^1*^

***Supplementary Table 1. Comparison of COVID-19 proportions by time period.***

^1^Chi-squared 2x3 P-value. ^2^Adjusting for Trust, age and sex.

***Supplementary Table 2. Sample characteristics by time period.*** Breakdown of total numbers (n) and percentage (%) of samples assayed according to age group, sex and Health and Social Care Trust across all three testing rounds.

|  | Round 1  June/July 2020 | Round 2  Nov/Dec 2020 | Round 3  June/July 2021 |
| --- | --- | --- | --- |
|  | n (%) | n (%) | n (%) |
| *Age group* |  |  |  |
| 0-29 | 74 (6.7%) | 152 (12.3%) | 341 (13.7%) |
| 30-49 | 185 (16.7%) | 278 (22.4%) | 585 (23.5%) |
| 50-69 | 394 (35.6%) | 452 (36.5%) | 850 (34.1%) |
| 70+ | 453 (41.0%) | 357 (28.8%) | 718 (28.8%) |
|  |  |  |  |
| *Sex* |  |  |  |
| Female | 580 (52.5%) | 682 (55.0%) | 1417 (56.8%) |
| Male | 525 (47.5%) | 559 (45.0%) | 1076 (43.2%) |
| *Health and Social Care Trust* |  |  |  |
| Belfast | 223 (20.1%) | 246 (19.8%) | 499 (20.0%) |
| Western | 210 (19.0%) | 248 (20.0%) | 495 (19.8%) |
| Southern | 225 (20.3%) | 248 (20.0%) | 500 (20.0%) |
| Northern | 225 (20.3%) | 250 (20.1%) | 500 (20.0%) |
| South Eastern | 225 (20.3%) | 250 (20.1%) | 500 (20.0%) |
